# Colombian *Anopheles triannulatus* (Diptera: Culicidae) Naturally Infected with *Plasmodium* spp.

**DOI:** 10.5402/2013/927453

**Published:** 2013-10-08

**Authors:** Doris A. Rosero, Nelson Naranjo-Diaz, Natalí Alvarez, Astrid V. Cienfuegos, Carolina Torres, Shirley Luckhart, Margarita M. Correa

**Affiliations:** ^1^Grupo de Microbiología Molecular, Escuela de Microbiología, Universidad de Antioquia, Calle 67 53-108, Lab 5-430, Medellín, Colombia; ^2^Linea de Entomología Médica, Programa de Estudio y Control de Enfermedades Tropicales (PECET), Universidad de Antioquia, Sede de Investigación Universitaria (SIU), Calle 62 no. 52-59, Lab 632, Medellín, Colombia; ^3^Department of Medical Microbiology and Immunology, University of California at Davis, Shields Avenue, Tupper Hall, Room 3146, Davis, CA, USA

## Abstract

The role of *Anopheles triannulatus* as a local vector has not yet been defined for malaria-endemic regions of Colombia. Therefore, the aim of this work was to detect *An. triannulatus* naturally infected with *Plasmodium* spp., as an approximation to determining its importance as malaria vector in the country. A total of 510 *An. triannulatus* were collected in six malaria-endemic localities of NW and SE Colombia from January 2009 to March 2011. In the NW, two specimens were naturally infected; one with *Plasmodium vivax* VK247, collected biting on humans and the other with *Plasmodium falciparum*, collected resting on cattle. In the SE, two specimens were positive for *P. falciparum*. Although these results show *An. triannulatus* naturally infected with *Plasmodium*, further studies are recommended to demonstrate the epidemiological importance of this species in malaria-endemic regions of Colombia.

## 1. Introduction

Malaria is an important public health problem in Colombia with approximately 60% of the territory being suitable for disease transmission [1]. In 2010, 116,914 malaria cases were reported [2], for an annual parasite index (API) of 11.58/1,000 inhabitants, and in 2011, only 62,969 cases were reported for API of 6.2/1,000 inhabitants [3], although underregistration of cases is presumed [4]. The Urabá-Bajo Cauca and Alto Sinú (UCS) epidemiological region, NW Colombia, registers near 60% of the total malaria cases of the country and the Amazonas region, SE, 0.67% [2, 5]. 

In Colombia, based on epidemiological, entomological or parasite detection records, at least nine *Anopheles *species have been incriminated as malaria vectors. Three are of primary importance: *Anopheles (Nyssorhynchus) albimanus* Wiedemann 1920, *Anopheles (Nys.) darlingi* Root 1926, and *Anopheles (Nys.) nuneztovari* Gabaldón 1940, and six other species have relevance as secondary or local vectors [6–9]. However, there are species that in neighboring countries have been incriminated as local or regional vectors, but their role in transmission is not yet established for Colombia [1]. This is the case of *Anopheles* (*Nys.*) *triannulatus* Neiva and Pinto 1922, a species widely distributed in the country [10]. Interestingly, in Cimitarra municipality, NE Colombia, it was the most abundant species collected and had an indoor biting behavior similar to the main vector, *An. nuneztovari* [11]. Also, in Santa Rosa de Lima, Bolivar, in northern Colombia, *An. triannulatus* was the predominant species, found in sympatry with the primary malaria vector *An. albimanus* [9]. Furthermore, in localities of the Amazon Region, SE Colombia, it was collected in peridomestic areas showing anthropophilic activity and in sympatry with the main vectors *An. darlingi* and *An. nuneztovari* [12]. Despite the preferentially zoophilic behavior reported for *An. triannulatus* in Brazil [13, 14], Colombia [9], and Venezuela [15], this species is suggested to play a role in malaria transmission as a secondary vector [16], especially, if it is in high densities [17], behaving as an opportunistic species depending on host availability and abundance [18]. In Brazil, *An. triannulatus* has been detected infected with* Plasmodium vivax *Grassi and Felleti 1890, in Pará State [18], Rondônia State [19, 20] and Amazonas State [21], with *Plasmodium falciparum* Welch 1897 and *Plasmodium malariae* Laveran 1880 in Amazonas [21] and Amapá State [17]. In other South American countries, *An. triannulatus* was reported as the dominant vector in eastern Loreto Department, Perú [22] and in Bolivia, and it is suggested to be a secondary vector [23, 24]. According to older reports, in Venezuela, *An. triannulatus* was epidemiologically implicated as a vector during a malaria outbreak [25], and some years later detected with natural oocyst infection [26], being considered a potential vector in western Venezuela where it showed a more anthropophilic behavior than *An. nuneztovari* and *An. oswaldoi* [27]. In Colombia, several studies have evaluated *An. triannulatus* for *Plasmodium* infection, using salivary glands and midgut dissection [28, 29] or ELISA and PCR [7–9, 30]; however, only recently, this species was detected infected with *Plasmodium vivax* [30].

*Anopheles triannulatus* is considered a complex of at least three species, *Anopheles triannulatus* s.s. Neiva and Pinto 1922, *Anopheles halophylus* Silva-do-Nascimento and Lourenço-de-Oliveira 2002, and *Anopheles triannulatus* C [14, 31], that may differ in their vectorial capacity and involvement in parasite transmission [32, 33]. Up to the present, *An. halophylus* and *An. triannulatus* C have not been incriminated in malaria transmission [14, 31]. In Colombia, there is no evidence for the existence of more than one species of the Triannulatus Complex. Only a recent study found a substantial genetic division, with two *An. triannulatus* lineages, NW and SE, coinciding with their geographical distributions in Colombia [34]. Consecutively, *An. triannulatus* NW lineage was detected infected with *P. vivax *in the NW [30]. This finding motivated a more comprehensive assessment of *An. triannulatus* parasite infection, through the evaluation of specimens collected in six localities of two malaria endemic regions, the Urabá-Bajo Cauca and Alto Sinú, NW, and the Amazonas region, SE Colombia. 

## 2. Materials and Methods

### 2.1. Mosquito Collection and Processing

**Figure 1:**
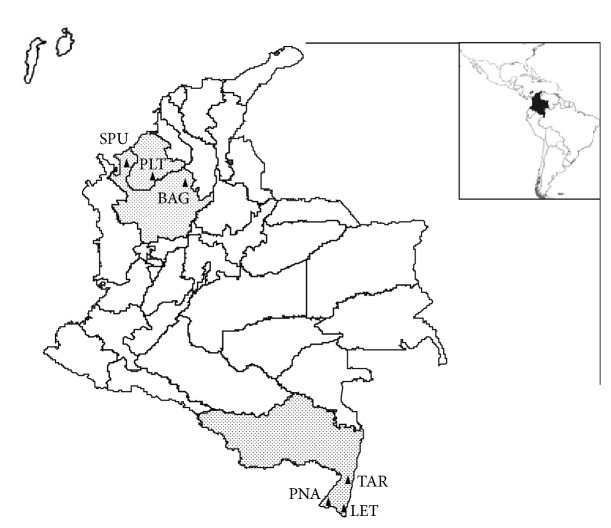
Localities of NW and SE Colombia where *Anopheles triannulatus* were collected. Puerto Libertador-PLT in Córdoba, El Bagre-BAG and San Pedro de Urabá-SPU in Antioquia, Tarapacá-TAR, Leticia-LET, and Puerto Nariño-PNA in Amazonas Department.

*Anopheles triannulatus* specimens were collected from January 2009 to March 2011 in six Colombian localities (Figure 1). These localities were selected based on the reports of malaria cases and *An. triannulatus* presence. The municipalities of Leticia-LET, Puerto Nariño-PNA, and Tarapacá-TAR in Amazonas Department, account for 70% of the malaria cases reported in the Amazonian region [35]. Antioquia and Córdoba Departments in the UCS region, NW, reported >60% of total number of malaria cases of the country in 2010 [2, 5]. Of these, three municipalities in Cordoba (Tierralta, Montelíbano, and Puerto Libertador) contributed with 18% and three in Antioquia (Bagre, Caceres and Zaragoza) with 42% of those cases [5]. Available data for some of the localities indicate that, in 2010 Bagre-BAG reported 14,258 cases and San Pedro de Urabá-SPU 1,040 [36]. Indoor and outdoor collections (within ~10 m of each house), from 18:00 to 24:00 h and at least one overnight (18:00–06:00 h) collection per field trip were performed, using human-landing catches under an informed consent agreement and collection protocol reviewed and approved by a University of Antioquia Institutional Review Board (Comité de Bioética Sede Investigación Universitaria, CBEIH-SIU, UdeA, approval document 07-41-082). In addition, in SPU and Puerto Libertador (PLT), NW, mosquitoes resting on cattle were collected in nearby corrals (within ~10 to 100 m from the house). At SE localities specimens were provided by trained personnel by means of a contract for mosquito collections, and only peridomestic collections from 18:00 to 23:00 h were conducted. All *Anopheles *specimens were identified based on morphological features [10]. Also, the species assignation for specimens with problematic morphology and for all *An. triannulatus* was confirmed by a PCR-RFLP-ITS2 assay [37, 38], and MegaBLAST of *An. triannulatus COI* sequences, accession numbers JX852142–JX852282 [34].

### 2.2. Detection of *Anopheles triannulatus *Naturally Infected with *Plasmodium* spp

The *An. triannulatus* specimens processed by Enzyme-linked immunosorbent assay (ELISA) were tested in pools of five head-thoraces grouped by collection date and locality. Detection of the circumsporozoite (CSP) protein specific for *P. falciparum *and *P. vivax* variants VK247 and VK210 was carried out in separate plates following procedures optimized by personnel of the Molecular Microbiology Group [8, 9]. ELISA positive specimens were those giving an OD result (WL: 405 nm) equal or above the cut off value, set as twice the mean of seven negative controls run consecutively in each plate. Positive pools were evaluated in a second ELISA, performed at a later date to reduce the chance of reporting false positives [9, 39]. Positive controls were those supplied with the ELISA test reagents. Lysates prepared from colonized *An. albimanus* served as negative controls.

For the nested PCR, DNA was extracted from individual abdomens following a salt precipitation protocol [40]. Amplifications using genus and species-specific primers designed to amplify the *Plasmodium* small ribosomal subunit DNA were performed using the primers and conditions previously described [41], with six *μ*L of extracted DNA used as template in a 25 *μ*L reaction mixture. For the genus nest-2 and the *Plasmodium* species-specific amplifications, six *μ*L of the previous amplification product served as the DNA template. *Plasmodium vivax* DNA was used as the positive control. Specimens with a positive nested PCR and ELISA result were further confirmed for *Plasmodium* infection using a PCR directed to detect a mitochondrial cytochrome b (*Cytb*) fragment specific for human *Plasmodium* spp. [42], using two *μ*L of extracted DNA as the template.

### 2.3. Data Analysis

Mosquito infection rates (IRs), or the percentage of *Plasmodium *infected* An. triannulatus*, were calculated as the number of positive *An. triannulatus* (np) per number of total analyzed (nt) per 100 [IR = (np/nt) × 100] [43]. The confidence interval (CI: 95%) was calculated to indicate the reliability of the estimated value using the EPIDAT program version 3.1 [44]. 

## 3. Results

### 3.1. *Anopheles triannulatus* Abundance

 Of a total of 510 *An. triannulatus* collected, 210 were from northwestern localities (Table 1). In BAG locality, 87 *An. triannulatus* were collected in four field trips and approximately 162 h of sampling, being the third most abundant species during the August and December 2009 collections (12% and 3%, resp.), after *An. darlingi* and *An. nuneztovari* (Table 1). In SPU, 39 *An. triannulatus* were collected during two field trips and 72 h of sampling. Here, it was also the third most abundant species (2%), after *An. darlingi* and *An. nuneztovari* in the February 2010 collection, and after *An. nuneztovari* and *Anopheles (Anopheles) punctimacula *s.l. in March 2011 (18%) (Table 1). It is worth noting that in PLT, after four collections with 168 h of sampling, *An. triannulatus* was only detected in the June 2010 collection, being the second most abundant species (28%) after *An. nuneztovari* (Table 1). Data on abundance and entomological parameters for other species collected in NW localities are available in [30]. 

In SE, a total of 300 *An. triannulatus* specimens were collected by HLC. In LET, 86 *An. triannulatus* were collected in two sites and approximately 15 h of sampling, being the most predominant species in both sites (63% and 80%) (Table 2). In PNA, 89 *An. triannulatus* were collected in three sites, during 60 h of sampling. This species was the predominant species during three samplings conducted in Puerto Rico site, PNA (58%, 97% y 83%) (Table 2). In TAR, 125 *An. triannulatus* were collected in six sites and 95 h of sampling. Remarkably, in San Sebastian, TAR, it was the second most abundant species (13%) after *An. darlingi* in the June 2010 collection and became the most predominant species (90%) during the October 2010 collection. However, in TAR, *An. darlingi* was the predominant species in the rest of the collections (Table 2). 

### 3.2. *Anopheles triannulatus* Infection by *Plasmodium* spp

**Figure 2:**
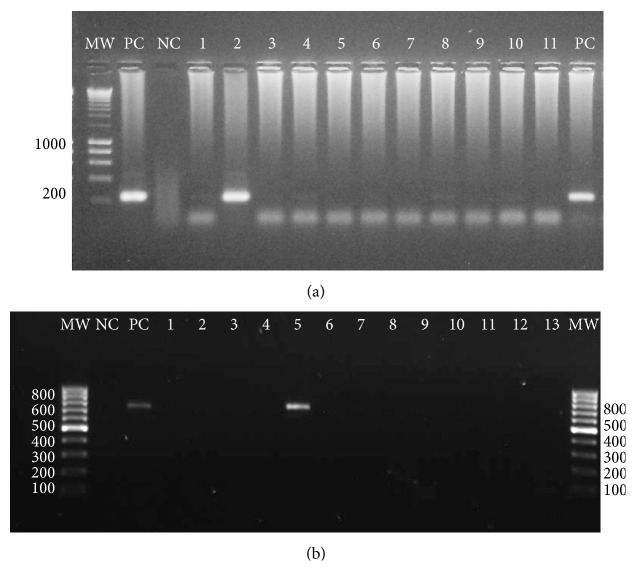
Nested and *Cytb* PCRs for *Plasmodium* detection in *An. triannulatus*. One % agarose gel. Lanes: MW: molecular weight, PC: Positive control—*Plasmodium vivax *DNA, NC: negative control (no DNA). (a). *Plasmodium* genus-specific Nested PCR. 1–11: DNA from *An. triannulatus* from Puerto Libertador (PLT), (b). PCR-*Cytb*, 1–13: *An. triannulatus* from various localities. Lane 5: positive specimen from PLT, equivalent to sample in lane 2 of gel (a).

In NW, two *An. triannulatus* specimens were detected with *Plasmodium* infection (Table 3), one in BAG infected with *P. vivax* VK247, as detected by only the first ELISA and the nested PCRs (*Plasmodium* and species-specific amplifications, IR = 1.51, CI: 0.04–8.15). This specimen was collected by HLC, indoors, between 18:00 to 19:00 h, during the January 2009 collection when *An. triannulatus* was found in low abundance (2%) (Table 1). In this locality, the main vector species were also detected infected: *An. nuneztovari* with *P. vivax* VK247 (IR = 0.10, CI: 0.003–0.559) and *An. darlingi* with *P. vivax* VK210 (IR = 0.087, CI: 0.002–0.485) [30]. Another *An. triannulatus* was detected infected with *P. falciparum* in PLT, NW, as determined by the nested PCRs, *Plasmodium* and species-specific amplifications (Figure 2(a)) and *Cytb *PCR (Figure 2(b)) (IR = 1.20; CI: 0.03–6.53); however, an ELISA positive result was not obtained (Table 3). This specimen was collected between 21:00 to 22:00 h, resting on cattle kept in corrals in proximity to the collection site.

In SE, two *An. triannulatus* collected by HLC were positive for *P. falciparum*, as detected by only the first ELISA, result that was confirmed by the nested genus-specific PCR (detects *Plasmodium*), however, the species-specific nested PCR or the *Cytb *PCR were negative (Table 3). One of the infected *An. triannulatus* was collected in Puerto Rico site, PNA (IR = 1.20, CI: 0.03–6.53), during the August 2010 sampling, when *An. triannulatus* was the most abundant species (97%) (Table 2). The other infected specimen (IR = 0.85, CI: 0.02–4.63) was collected in Ventura site, TAR, in June 2010, when *An. triannulatus* was the second most abundant species (20.3%) (Table 2). 

## 4. Discussion

The determination of parasite infection in anopheline mosquitoes is an important component for vector incrimination [45]. In Colombia, several studies have attempted to elucidate the role of *An. triannulatus* in transmission [7–9, 30], but, only recently, *An. triannulatus* NW linage [34] was reported infected with *P. vivax* VK247 [30]. In this study, a more thorough evaluation of *An. triannulatus* infection in specimens collected at various localities of malaria endemic areas of the country provided further evidence of *Plasmodium*-infected* An. triannulatus*. In BAG, NW, *An. triannulatus* collected biting in humans was generally in low abundance. In this locality, the specimen found infected had *P. vivax* [30], and the IR was of 1.51. The IRs for *An. triannulatus* infected with *P. falciparum* ranged from 0.85 to 1.20. These IR values are similar or slightly higher than those found for the main Colombian vectors, *An. albimanus* in the Colombian Pacific region [9], and in the same locality, BAG, *An. nuneztovari* was detected infected with *P. vivax* VK247 and *An. darlingi* with *P. vivax* VK210 [30]. These IRs are also similar to the ones previously found for *An. triannulatus* in Brazil, where it was detected infected with the same *Plasmodium* species. For example, with *P. vivax*, IR was 0.5 [19] and 1.1 [20] in Rondônia State; with *P. falciparum*, IR was 0.8 in Amazonas State [21] and 0.13 in Amapá State [17]. However, in some sites of Brazil, higher IRs have been detected for *An. triannulatus* infected with *P. vivax*, of 8.9 in Pará State [18] and 3.7 in Amazonas State [21]. 

The infected *An. triannulatus* from BAG, NW, was collected indoors, which makes it even more important to further evaluate the epidemiological role of this species in this locality. Interestingly, a previous work in various localities of this region showed *An. triannulatus* with anthropophilic tendency and active indoors and outdoors [8]. However, the infected *An. triannulatus* collected resting on cattle in PLT, NW, suggests a zoophilic tendency, as previously documented for this species in this particular area, where one of the main economic activities is livestock production and the high availability of cattle may influence this feeding behavior [8]. The two specimens detected infected in PNA and TAR localities of the Amazonian region, SE Colombia, had *P. falciparum*. These infected specimens were collected in periods when *An. triannulatus* was the most or second most abundant species in PNA and TAR, constituting 97% and 20% of the total collected specimens, respectively; notably in PNA, the primary vector *An. darlingi* was not detected in this collection period. This observation is similar to reports for *An. triannulatus* in localities of Pará, and Amazonas, Brazil, that indicate that this species colonized altered environments and became abundant and even dominated, outnumbering the main vector *An. darlingi* [21]. Of particular interest, PNA and TAR are in close proximity to LET (SE), a locality also included in a recent phylogeography study on *An. triannulatus *s.l. which evaluated specimens along the known geographic distribution of the species [46]. Of seven lineages proposed based on *COI* data, four were present in Colombia; particularly, lineage E, widely distributed in Brazil, where *An. triannulatus* s.l. has been found infected with *Plasmodium* spp. [17, 21], is also present in LET, SE; this makes it important to further investigate the role of *An. triannulatus* in transmission in these SE localities.

In this study, *An. triannulatus* was detected infected with both *P. vivax *and* P. falciparum*; however, only until recently this species was reported infected [30]. Among the reasons influencing the negative results could be that *An. triannulatus* were at low abundance [8] or not being participating in transmission when collections were carried out. Moreover, laboratory experiments have indicated that *An. triannulatus* is less susceptible to salivary gland sporozoite infection than *An. darlingi* or other vectors [47]. Also, technical reasons can produce negative results; for example, in the case of low parasite infection in the mosquito, the ELISA could show false negative results because of the lower sensitivity of this technique as compared to the PCR [43, 48, 49]. Furthermore, negative results could originate from the specimen part used for the test in relation to the stage of the parasite cycle in the mosquito; for example, at the initial stage of sporogony when the sporozoites have not reached the salivary glands [18, 50], the CSP protein could be detected only in the midgut [51]. In the case of the PCR, interfering material present in the reaction and a low DNA concentration after extraction could produce a false negative result [52]. Likewise, false positive results of the test to detect *Plasmodium*-infected anophelines have been reported; specifically for the ELISA, when testing zoophilic mosquitoes [39]. Therefore, this should be a consideration when analyzing ELISA positive *Plasmodium* infection results in *An. triannulatus*, especially in NW localities where cattle is a main mosquito-feeding source. Therefore in this study, to avoid reporting false positive results, at least two positive tests of the ones performed (two ELISA reactions, *Plasmodium* genus and species-specific nested PCRs or *Cytb* PCR), served as criteria for defining *Plasmodium* infected *Anopheles*. In the case of the *P. falciparum*-infected *An. triannulatus* collected in cattle in PLT, NW, the positivity was determined by both nested and *Cytb *PCR. For the other infected specimens, a positive result was obtained in the first ELISA and confirmed by nested PCR. In accordance with our findings, variations in the results of the tests used to detect mosquito parasite infection have been reported in other studies [52, 53]. 

## 5. Conclusions

In this study, *An. triannulatus* was detected infected with both *P. vivax *and* P. falciparum*, which suggests the importance of further evaluating the epidemiological importance of this species in malaria endemic regions of Colombia. In addition, differences in results between the PCR and ELISA advocate the need for assessing the degree of sensitivity and specificity of these two techniques. But even of greater priority is the evaluation or development of new, more accurate methodologies to test *Plasmodium* infection in the *Anopheles* mosquitoes. In this sense, the novel PCR based on parasite *Cytb* sequences [42], rapid test as the VecTestTM dipstick assay [54, 55], and real-time PCR amplification [56, 57] deserve further evaluation. While this is done and given the limitations still found when using the available methodologies, it is recommended that at least two positive tests be used as the criterion for defining *Plasmodium* infection in wild collected *Anopheles*. This is of particular importance when investigating the role of presumptive vector species in disease transmission. 

## Figures and Tables

**Table 1:** *Anopheles* species composition and proportion of *Anopheles triannulatus* from NW Colombia, collected January 2009–March 2011.

Department, locality	Year, month (no. of days)	Number of *Anopheles *collected	*An. triannulatus *s.l. no. (%)	Predominant species (%), others (%)^f^
Antioquia				
(i) El Bagre-BAG, La Capilla	2009, January (6)∗	585	12 (2%)^a^	*An. darlingi *(62%),
				*An. nuneztovari *s.l. (28%),
				others (8%)^f^
	2009, April (2)–May (4)	964	13 (1%)^b^	*An. nuneztovari *s.l. (50%),
				*An. darlingi *(43%),
				others (6%)^f^
	2009, August (6)	403	50 (13%)^b^	*An. nuneztovari *s.l. (57%),
				*An. darlingi *(25%),
				others (5%)^f^
	2009, December (5)	380	12 (2%)	*An. darlingi *(63%),
				*An. nuneztovari *s.l. (33%),
				others (2%)^f^
	Total	**2,332**	**87**	
(ii) San Pedro de Urabá-SPU, El Caño	2010, February (5)	589	14 (2%)^c^	*An. darlingi *(67%),
				*An. nuneztovari *s.l. (25%),
				*An. albimanus* (5%),
				*An. pseudopunctipennis *s.l. (0.5%),
				*An. punctimacula *(0.5%)
	2011, March (5)	143	25 (18%)^d^	*An. nuneztovari *s.l. (34%),
				*An. punctimacula *(32%),
				*An. albimanus* (14%),
				*An. albitarsis *s.l. (1%),
				*An. darlingi *(1%)
	Total	**732**	**39**	

Córdoba				
Puerto Libertador-PLT, Juan José	2009, July (1)–Aug (5)	2070	0	*An. nuneztovari *s.l. (99%),
				*An. darlingi *(1%)
	2009, November (6)	121	0	*An. nuneztovari *s.l. (99%),
				*An. darlingi *(1%)
	2010, February (6)	1068	0	*An. pseudopunctipennis* (73%),
				*An. nuneztovari *s.l. (25%),
				others (2%)^f^
	2010, June (6)^†^	302	84 (28%)^e^	*An. nuneztovari *s.l. (70%),
				*An. darlingi *(1%),
				others (1%)^f^
	Total	**3,561**	**84**	

Alternative site of collection for *An. triannulatus*: resting on the walls of the house: ^a^four, ^b^one, ^c^13; resting on cattle: ^d^21, ^e^69, ^f^reported in Naranjo-Diaz et al., 2013 [30]. ∗Infection rate (IR): 1.51, CI: 0.04–8.15/with *P. vivax* VK247, collected by HLC, indoors, between 18:00 and 19:00 h. ^†^IR: 1.20; CI: 0.03–6.53/with *P. falciparum*, collected between 21:00 and 22:00 h, resting on cattle kept in corrals in proximity to the collection site.

**Table 2:** *Anopheles* species composition and proportion of *Anopheles triannulatus* from SE Colombia, collected October 2009–June 2010.

Municipality/locality	Year, Month (no. of days)	Number of *Anopheles *collected	*An. triannulatus *s.l. no. (%)	Predominant species (%), others (%)
Leticia-LET				
(i) Km11	2009, October (2)	65	41 (63%)	*An. darlingi *(23%), others (14%)
(ii) Km6	2009, October (1)	56	45 (80%)	Others (16%), *An. darlingi* (4%)
	Total	**121**	**86**	
Puerto Nariño-PNA				
(i) 12 de Octubre	2009, September (1)	28	0%	*An. darlingi* (100%)
	2010, Aug-Sep (2)	4	3 (75%)	Others (25%)
(ii) Puerto Rico	2009, September (1)	24	14 (58%)	*An. darlingi* (25%), others (17%)
	2010, August (3)^*^	33	32 (97%)	Others (3%)
(iii) San Juan del Soco	2010, September (4)	47	39 (83%)	Others (17%)
	2009, September (1)	33	1 (3%)	*An. darlingi* (97%)
	Total	**169**	**89**	
Tarapacá-TAR				
(i) Comunidad San Sebastián	2010, Jun-July (6)	198	25 (13%)	*An. darlingi* (83%), others (4%)
(ii) Nueva Unión	2010, October (4)	48	42 (89%)	Others (9%), *An. darlingi* (2%)
(iii) Puerto Nuevo	2010, June (2)	55	1 (2%)	*An. darlingi* (80%), others (18%)
(iv) Quinina	2010, June (2)	50	9 (20%)	*An. darlingi* (56%), others (24%)
(v) Santa Lucía	2010, June (1)	36	5 (17%)	*An. darlingi* (83%)
(vi) Ventura	2010, June (2)	117	31 (28%)	*An. darlingi *(70%), others (2%)
	2010, June (2)^†^	59	12 (20%)	*An. darlingi *(71%), others (9%)
	Total	**563**	**125**	

^*^Infection rate (IR): 1.20, CI: 0.03–6.53/with *P. falciparum*. ^†^IR: 0.85, CI: 0.02–4.63/with *P. falciparum*. Collected by HLC between 18:00 and 19:00 h.

**Table 3:** Natural infection on *Anopheles triannulatus* from Northwestern and Southeastern Colombia.

Region/locality/collection site	No. of *An. triannulatus *analyzed	*Plasmodium *species^a^: infection rates^b^ (95% CI)	ELISA test		PCR	
			First	Second	Nested	*Cytb *
				(confirmation)		
NW						
El Bagre-BAG, La Capilla	82	*Pv*VK247: 1.51 (0.04–8.15)	Positive	Negative	Positive	Negative
Puerto Libertador-PLT, Juan José	83	*Pf*: 1.20 (0.03–6.53)	Negative	Negative	Positive	Positive
SE						
Puerto Nariño (PNA), Puerto Rico	83	*Pf:* 1.20 (0.03–6.53)	Positive	Negative	Positive∗	Negative
Tarapaca (TAR), Ventura	118	*Pf:* 0.85 (0.02–4.63)	Positive	Negative	Positive∗	Negative

The table shows only results for those localities where naturally infected *An. triannulatus* were detected. ^a^ *Plasmodium *species:* Pv*VK247*-P. vivax *VK247*, Pv*VK210*-P. vivax *VK210*, Pf: P. falciparum*.^b^Number of positive *An. triannulatus* (np) per number of total analyzed (nt) per 100, determined for each locality, (IR = (np/nt) × 100). ∗Positive result only in the nested PCR that detects *Plasmodium *genus, negative in the nested PCR that detects *Plasmodium* species. Due to the limitations on the available tests for detecting naturally infected anopheline mosquitoes (see discussion), positive specimens were determined as those given a positive result in at least two of the tests.
